# Histomorphological and functional contralateral symmetry in the gastrocnemius muscles of the laboratory rat

**DOI:** 10.1111/joa.13674

**Published:** 2022-04-18

**Authors:** Garoa Santocildes, Marc Merino, Federica Fabiani, Teresa Pagès, Mario Marotta, Ginés Viscor, Joan Ramon Torrella

**Affiliations:** ^1^ Departament de Biologia Cel·lular Fisiologia i Immunologia, Universitat de Barcelona Barcelona Spain; ^2^ Vall d'Hebron Institut de Recerca, Universitat Autònoma de Barcelona Barcelona Spain

**Keywords:** fibre types, histochemistry, laterality, muscle force

## Abstract

It is usual in anatomical and physiological research to assess the effects of some intervention on extremities (e.g., training programmes or injury recovery protocols) using one muscle for the intervention and its contralateral as control. However, the existence of laterality (left‐handedness or right‐handedness) in athletes of different specialities is widely recognized. In rats, gastrocnemius is one of the muscles most widely used because of its importance in locomotion and high relative limb mass. Since we have not found studies reporting laterality assessment on the morphology and function in rat gastrocnemius, our study aimed to evaluate the fibre histochemical, morphometrical and muscle force contractile properties between right and left gastrocnemius of the laboratory rat. Fibre‐type proportion, fibre morphometrical measurements, muscle capillarization and muscle force properties were analysed in the right and left gastrocnemius of six male rats. No statistically significant differences (*p* = 0.265) were found in gastrocnemius to body weight ratio (‰) between right (6.55 ± 0.40) and left (6.49 ± 0.40) muscles. The muscles analysed showed a great degree of heterogeneity in fibre type distribution, having three clearly distinguished regions named *red*, *mixed* and *white*. In the three regions, there were no statistical differences in fibre type proportions between right and left gastrocnemius, as is indicated by the *p*‐values (from 0.203 to 0.941) obtained after running *t*‐Student paired tests for each fibre type. When analysing fibre cross‐sectional area, individual fibre capillarization and fibre circularity, no significant differences between right and left gastrocnemius in any of these morphometrical parameters were found in any muscle region or fibre type. Most of the *p*‐values (70%) resulting from running *t*‐Student paired tests were higher than 0.400, and the lowest *p*‐value was 0.115. Seemingly, global capillary and fibre densities were not statistically different between right and left sides in all muscle regions with *p*‐values ranging from 0.337 to 0.812. Force parameters normalized to gastrocnemius mass (mN g^−1^) did not show any significant difference between right (PF = 74.0 ± 13.4, TF = 219.4 ± 13.0) and left (PF = 70.9 ± 10.7, TF = 213.0 ± 18.0) muscles with *p* = 0.623 (PF) and *p* = 0.514 (TF). Twitch time parameters (ms) also lacked significant differences between the two sides (CT: 43.4 ± 8.6 vs. 45.0 ± 14.3, *p* = 0.639; HRT: 77.6 ± 15.0 vs. 82.3 ± 25.3, *p* = 0.475). Finally, both muscles also showed similar (*p* = 0.718) fatigue properties. We did find an absence of laterality at the morphological and functional levels, which raises the possibility of using right and left gastrocnemius muscles interchangeably for experimental designs where one muscle is used to analyse data after a physiological intervention and its contralateral muscle plays the control role, thus allowing unbiased paired comparisons to derive accurate conclusions.

## Graphical abstract

Our results report the absence of differences between right and left sides in muscle morphology (size and proportion of fibre types and muscle capillarization) and muscle functional properties (twitch and tetanic force, twitch time parameters, fatigue parameters and force‐frequency curves).

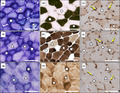

## 1 INTRODUCTION

The unilateral leg model consists of the intervention of one leg or muscle group (e.g., subjected to exercise or ischemia, electrostimulated, injured or denervated), while the contralateral leg or muscle is used as an internal control (Contreras‐Muñoz et al., 2017; Contreras‐Muñoz et al., 2021; Masiero et al., 2009; Pereira et al., 2014). This experimental approach has been extensively used in the field of muscle morphological and physiological research (Guo & Zhou, 2003; Lexell & Taylor, 1991; Rab et al., 2000; Tarnopolsky et al., 2007), and is based on the assumption that, prior to the intervention, both legs or muscle pairs displayed similar morphological and physiological properties regarding the outcome of interest (Tarnopolsky et al., 2007) and on the assumption that the unilateral intervention has no impact on the contralateral leg.

Animals´ muscle symmetry between the right and left sides has been widely accepted from the morphological point of view. In fact, even reference works on muscle morpho‐histology only include the muscles from one side of the body in their experimental design and analysis (Armstrong & Phelps, 1984). The similarity of the contralateral muscles has been also assumed when the influence of different factors was studied on the complex 3D muscle architecture. For example, Wick et al. (2018) compared the muscle architecture of one isolated muscle versus its contralateral muscle surrounded by adjacent muscles, and Schenk et al. (2020) examined the influence of joint angle on 3D muscle architecture by comparing short left muscle and long right muscle. Obviously, these investigations are valid only if the right and left muscles were almost similar. In addition, in most studies, biochemical and physiological determinations are only performed on the muscles from one side of the sagittal line, considering that the paired muscle has a similar profile (Cornachione et al., 2011; Panisello et al., 2008; Rizo‐Roca et al., 2017; Rizo‐Roca et al., 2018). Thus, considering the scarcity of experimental results, it is probably inappropriate to affirm that they are free of laterality (Guo & Zhou, 2003). However, at least humans show marked laterality or predisposition to preferential use in voluntary motor actions of parts of one side of the body over the other (Carpes et al., 2010; Kertesz et al., 1992). It is known that the asymmetric use of muscles can drive structural changes (Adam et al., 1998; Fugl‐Meyer et al., 1982; Lexell & Taylor, 1991). Some studies have reported differences between contralateral muscles of human upper extremities, both on fibre type proportion and strength, these changes being dependent on the use and functional demands of each particular muscle (Adam et al., 1998; Aoki & Demura, 2008; Fugl‐Meyer et al., 1982; Kubota & Demura, 2011; Venturelli et al., 2021). Regarding lower limbs, since they are usually used for symmetrical actions, less pronounced laterality than in upper limbs is observed (Maupas et al., 2002). There are very few studies investigating whether there is morphological or physiological symmetry between the muscles of the right and left parts of the lower body (Horwarth et al., 2021; Lexell & Taylor, 1991; Tarnopolsky et al., 2007).

For methodological and ethical reasons, the laboratory rat is one of the most widely used species in scientific research (Andersen & Tufik, 2016). An obvious question, which has been posed a few times in scientific literature, arises: could there be any structural, metabolic or functional difference between rats' right and left muscles? Among all rat muscles, the gastrocnemius is one of the most widely used in biomedical research due to its important role in locomotion and its high relative leg mass (Armstrong & Phelps, 1984; Cornachione et al., 2011). Moreover, its superficial location allows easy access for intervention, isolation and dissection. The aim of our study was to elucidate if there are morpho‐functional differences between the right and left rat gastrocnemius muscles. The results will allow us to conclude if the experimental design of the unilateral leg model is correct and if the muscles from both sides can be used interchangeably.

## 2 METHODS

### 2.1 Animals

Fourteen male (9‐week‐old) Sprague–Dawley rats with a body mass of 289 ± 8 g (mean ± SD) were used in the study. Animals were housed at 25 ± 2°C and maintained on a 12‐h light–dark cycle, with ad libitum access to water and food. The protocol was performed following the European Union guidelines for the care and management of laboratory animals and the Spanish Law on Animal Protection under license from the Catalan authorities (reference no. 1899), as approved by the University of Barcelona's Ethical Committee for Animal Experimentation.

### 2.2 Force measurements

After a week of quarantine, an in vivo determination of the gastrocnemius muscle contractile properties was performed. Animals were anaesthetized by an intraperitoneal injection of ketamine (75 mg/kg) and xylazine (10 mg/kg) and placed in a prone position on a dissection platform. Ketamine‐xylazine anaesthetics were used following the current regulations by the local institutional Ethical Committee for Animal Experimentation (CEEA number 1899) and used in previous similar studies (Contreras‐Muñoz et al., 2017, 2021). The gastrocnemius muscle was isolated from the surrounding musculature, leaving intact the blood supply and the nerves of the proximal insertion. The hind limb was immobilized by securing the knee and the ankle to the dissecting platform. The sciatic nerve was then exposed through a lateral incision on the thigh and connected to an electrode stimulator. Finally, the calcaneus tendon was cut at the distal insertion and tied to a force transducer (MLT 1030/D; ADInstruments) with an initial tension of 30 mN. To maintain optimal contraction conditions, the muscle was covered with a mineral oil solution (Sigma‐Aldrich), which prevented it from drying out, and muscle temperature was maintained with a heat lamp (Daylight Basking Spot lamp 50 W; Exo Terra) which placed at 20 cm heated 33°C, according to manufacturer's specifications. Muscle temperature was checked at the beginning, during and at the end of the force measurement protocols by means of a non‐contact infrared surface thermometer (PCE FIT‐10; Spain), registering muscle temperature within a narrow range of variation (32–35°C). It has been described that this temperature range does not interfere with the muscle contractile properties (Ranatunga, 1982). Muscle contraction was elicited via supramaximal electrical sciatic nerve stimulation (3 mA, pulse width 0.05 ms) with the Stimulus isolator FE180 (ADInstruments). To set up the supramaximal response, the pulse width was fixed at 0.05 ms (according to manufacturer specifications) and the maximal twitch response was determined by increasing the intensity of the stimulus' current from 0.1 mA until further increases in stimulator intensity produced no further increase in the twitch amplitude. To ensure a supramaximal stimulation, the intensity producing the maximal response was multiplied by a factor of 1.5, resulting in 3 mA.

Before carrying out the different force measurements, the gastrocnemius muscle's optimal contraction length, at which its maximum twitch force is produced, was set with a series of twitch contractions. After that, the following stimulation protocol was applied to measure several muscle function parameters: (1) five isometric muscle twitches were recorded at a frequency of 1 Hz and the average of five consecutive isolated twitches was considered to obtain a single twitch peak force (PF, in mN), contraction time (CT, in ms) and half‐relaxation time (HRT, in ms); (2) train of stimuli during 1 s at frequencies of 10, 20, 30, 40, 50, 60, 70, 80, 90, 100, 150 and 200 Hz (with pauses of 1 min between trains to avoid muscle fatigue) were given to obtain force‐frequency curve and the maximum tetanic force (TetF, in mN): (3) after 5 min of recovery (Allen et al., 2008), a fatigue test at low‐frequency during 2 min was applied by continuous muscle stimulation at a frequency of 40 Hz and a fatigue index was calculated by measuring the area relative to baseline (force‐time fatigue, in N s); and (4) step 1 was repeated and the percentage of post‐fatigue PF with respect to the initial PF was calculated. The stimulation protocol was carried out on both gastrocnemius muscles of each rat, the order in which left and right muscles were tested was randomized, and the force recordings were normalized to the muscle mass. To measure and analyse all the parameters, the AD Instruments hardware (PowerLab/16SP) and software (LabCHart v7.3.7) were used. At the end of the force test, both gastrocnemius muscles were excised, weighed, and frozen at their resting sarcomere length in pre‐cooled isopentane (Sigma‐Aldrich) and finally stored at −80°C until further analysis.

### 2.3 Histochemical procedures

Serial transverse cross‐sections (14–16 μm) from the equatorial regions of the gastrocnemius muscle at 7 mm from the end of the distal myotendinous junction were perpendicularly cut in a cryostat at −20°C (Leica CM3050S). Muscle samples were placed in an orthogonal angle with respect to the fibres' orientation. Adjacent serial sections were mounted on gelatinized slides (0.02%) and processed with different histochemical techniques. First, all the sections were fixed for 5 min in formalin‐sucrose solution to prevent shrinkage or wrinkle of the samples, and then they were incubated to: (1) succinate dehydrogenase staining (SDH), to recognize aerobic and anaerobic fibres (Nachlas et al., 1957), (2) alkaline myosin adenosine triphosphate (mATPase) after alkaline pre‐incubation (pH 10.7), to differentiate between slow and fast‐twitch fibres (Brooke & Kaiser, 1970), and (3) endothelial adenosine triphosphate (eATPase), to detect muscle capillaries (Fouces et al., 1993).

### 2.4 Fibre typing, morpho‐functional measurements and capillary count

All muscle fibres were typified according to their metabolic and contractile profile and classified as: (1) slow‐twitch oxidative (SO), (2) fast‐twitch oxidative glycolytic (FOG), (3) fast‐twitch glycolytic (FG) or (4) fast‐twitch intermediate glycolytic (FIG). SO fibres had no mATPase activity (pH 10.7) and showed high SDH staining; FOG fibres demonstrated both high mATPase and SDH staining; FG fibres presented moderate mATPase staining and showed very low SDH staining; FIG fibres presented from moderate to high mATPase staining and intermediate SDH (higher than FG and lower than FOG).

**FIGURE 1:**
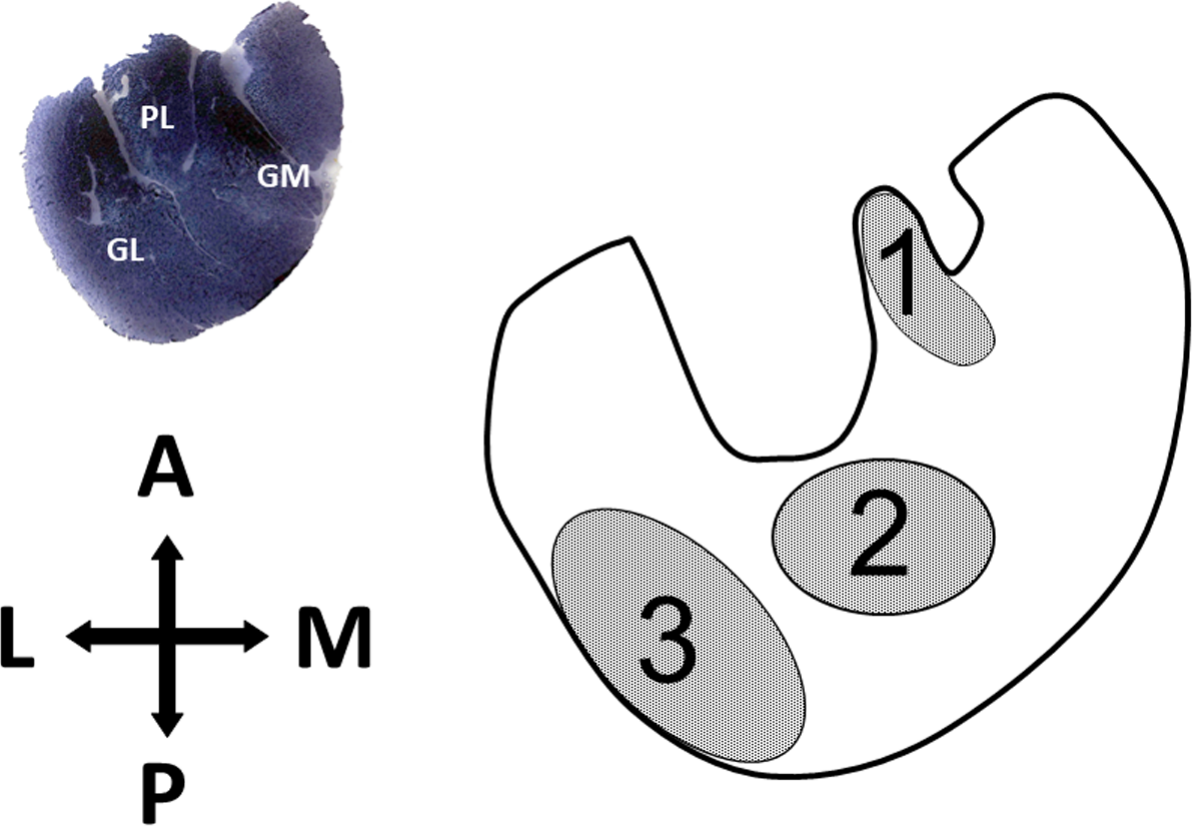
Schematic representation of the transverse section of the gastrocnemius muscle and the regions studied. A, anterior; GL, gastrocnemius (lateral); GM, gastrocnemius (medial); L, lateral; M, medial; P, posterior; PL, plantaris muscle. Numbers are gastrocnemius regions: 1 (red), 2 (mixed), 3 (white)

Microphotographs of stained muscle sections were obtained with a light microscope (BX61; Olympus) connected to a digital camera (DP70; Olympus) at 20× magnification. Three different regions of the gastrocnemius muscle (Armstrong & Phelps, 1984) (Figure 1) were considered for analysing the parameters listed below, measured or calculated from images with an area of 5.5·10^5^ μm^2^ using ImageJ software (v. 1.51n; National Institute of Health, USA). Images from eATPase were used to measure fibre cross‐sectional area (FCSA, μm^2^), fibre circularity shape factor (SF = 4πFCSA/perimeter^2^) and for calculating the number of capillaries per 1000 μm^2^ of FCSA (CCA), fibre density (FD) and capillary density (CD) per mm^2^.

### 2.5 Statistical analysis

Statistical analysis was performed using the statistical package SigmaPlot 11 (Systat Sofware, Inc., 2008–2009). Statistical comparisons were made between left and right gastrocnemius by paired Student *t‐*tests where *p* < 0.05 was considered significant. One‐way ANOVA tests were used for comparisons between parameters from different fibre types and Holm‐Sidak multiple tests were run as post hoc pairwise comparisons. Figures are presented as box‐and‐whisker plots. The box being the interquartile range and shows the second and the third quartiles separated by the median. Whisker endpoints represent the minimum and maximum values and the mean is indicated in the box with a black dot.

## 3 RESULTS

### 3.1 Fibre types

**FIGURE 2:**
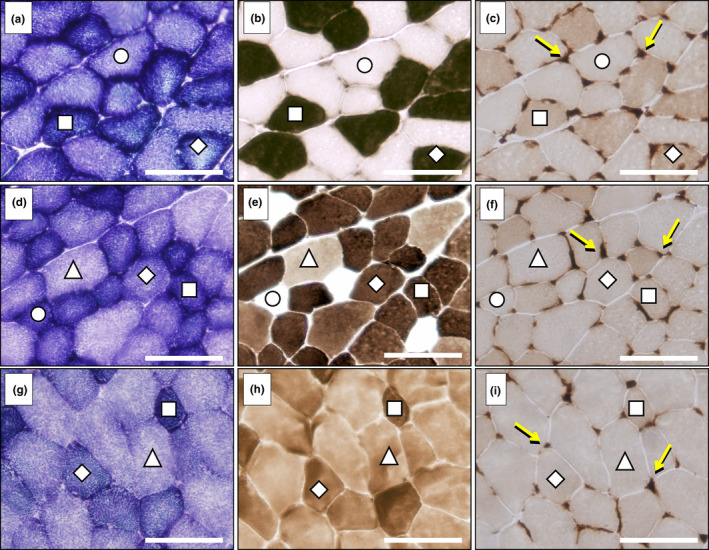
Fibre types in the red (a–c), mixed (d–f) and white (g–i) regions of the rat gastrocnemius muscle. Serial transverse muscle sections were stained for: (a, d, g) succinate dehydrogenase; (b, e, h) myosin ATPase after alkaline pre‐incubation (pH 10.7); (c, f, i) endothelial ATPase (arrows indicate muscle capillaries). Fibre types were classified as: FG, fast glycolytic (Δ); FIG, fast intermediate glycolytic (◊); FOG, fast oxidative glycolytic (□); SO, slow oxidative (○). Scale bar, 100 μm

**FIGURE 3:**
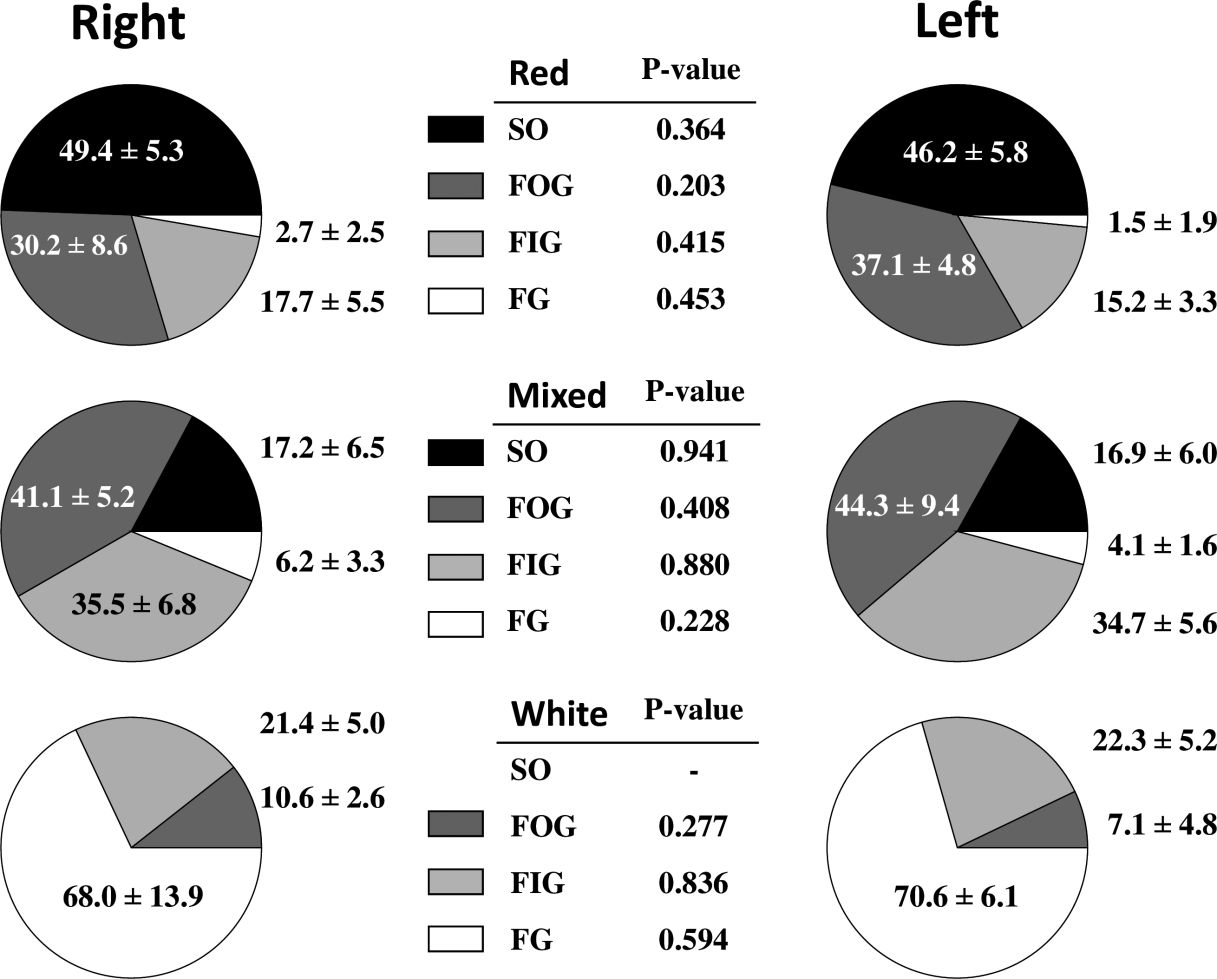
Pie charts showing the percentage of fibre type distribution in red, mixed and white regions of the right and left rat gastrocnemius muscles. *p*‐values resulting from statistical comparisons between right and left muscles after running paired *t*‐Student tests are shown for each fibre type in the central tables. Values are mean ± standard deviation. FG, fast glycolytic; FIG, fast intermediate glycolytic; FOG, fast oxidative glycolytic; SO, slow oxidative

The gastrocnemius muscles analysed in our study showed a great degree of heterogeneity in fibre type distribution, having three clearly distinguished regions named *red*, *mixed* and *white*. The histochemical assays used for demonstrating a fibre's oxidative character and mATPase activity after alkaline pre‐incubation made it possible to identify four fibre types that were unevenly distributed throughout the three analysed regions of the gastrocnemius. Figure 2 shows representative microphotographs of the three gastrocnemius regions with indications of the four fibre types' profiles and Figure 3 displays the percentages of fibre type distributions throughout the right and left muscles. The red zone is a highly oxidative region with only a residual percentage of FG fibres in some animals, this fibre type being absent in most individuals. It is noteworthy that this region consisted of almost 50% of SO fibres. The mixed region is the most heterogeneous, having the four fibre types in all the muscles analysed and showing a predominance of fast oxidative fibres (FOG and FIG). The white region presented only fast fibres in which the anaerobic type (FG) prevailed. In the three regions, there were no statistical differences in fibre type proportions between right and left gastrocnemius, as is indicated by the *p*‐values (from 0.203 to 0.941) obtained after running *t*‐Student paired tests for each fibre type (Figure 3).

### 3.2 Fibre morphometry

**FIGURE 4:**
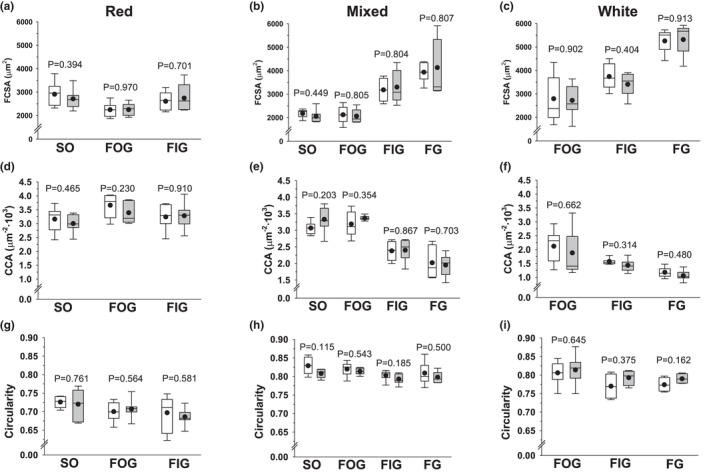
Morphometry of the different fibre types in red, mixed and white regions of the right and left rat gastrocnemius muscles. (a–c) fibre‐cross sectional area (FCSA); (d–f) number of capillaries per 1000 μm^2^ of fibre cross‐sectional area (CCA); (g–i) circularity shape factor. The boxes represent the first and third quartiles separated by the median, the mean is represented with a black dot, and the whisker end points represent the minimum and maximum values. Data from the right gastrocnemius are shown in white boxes and data from the left gastrocnemius in grey boxes. *p*‐values resulting from statistical comparisons between right and left muscles after running paired *t*‐Student tests are shown for each fibre type on the graphs. FG, fast glycolytic; FIG, fast intermediate glycolytic; FOG, fast oxidative glycolytic; SO, slow oxidative

Figure 4 presents the box plots showing the fibre sizes (fibre cross‐sectional area, FCSA), the individual fibre capillarization expressing the number of capillaries per 1000 μm^2^ of fibre area (CCA), and the shape factor (SF) estimating the circularity of the fibres (SF = 1 meaning a perfect circle). We decided to maintain the same axis scales within the same parameter to allow morphometrical comparisons between the three gastrocnemius regions. There were no significant differences between right and left gastrocnemius in any fibre morphometrical parameter from any region or fibre type. Most of the *p*‐values (70%) resulting from running *t*‐Student paired tests were higher than 0.400, and the lowest *p*‐value was 0.115.

A gradual increase in fibre size from oxidative fibres (smallest) to intermediate and anaerobic fibres (greatest) is evident in mixed and white regions (Figure 4b,c), yielding significant differences among fibre types after running one‐way ANOVA tests (*p* < 0.001). In the mixed region (Figure 4b), pairwise multiple comparison post hoc tests established no statistical differences between SO and FOG but significant differences between both SO and FOG versus FIG and FG (*p* < 0.001 in all pairwise comparisons) and between FIG and FG (*p* = 0.003). In the white region (Figure 4c), the post hoc tests showed *p* < 0.001 values when comparing FG versus FOG or FIG and *p* = 0.010 in the comparison of FOG versus FIG. However, in the red region (Figure 4a) FOG had significantly smaller FCSA than SO (*p* < 0.001) and FIG (*p* = 0.010), SO and FIG being similar in size.

The individual fibre capillarization (CCA) showed the same behaviour as FCSA, with a greater number of capillaries per fibre area in the more oxidative fibres in mixed and white regions (Figure 4e,f). In the mixed region (Figure 4e), pairwise multiple comparison post hoc tests established no statistical differences between SO and FOG but significant greater values for both SO and FOG than for FIG and FG (*p* < 0.001 in all pairwise comparisons) and greater values for FIG than for FG (*p* = 0.006). In the white region (Figure 4f), the post hoc tests showed *p* < 0.001 values when comparing FG versus FOG, *p* = 0.040 versus FIG and *p* = 0.011 in the comparison of FOG versus FIG. In the red region (Figure 4d), the only significant pairwise difference was between SO and FOG fibres (*p* = 0.008).

The only significant difference in circularity was found between SO and FOG fibres (*p* = 0.008) in the red region (Figure 4g), since no significant differences were found in either the mixed or the white region between fibre types (Figure 4h,i).

### 3.3 Global regional capillary and fibre densities

**FIGURE 5:**
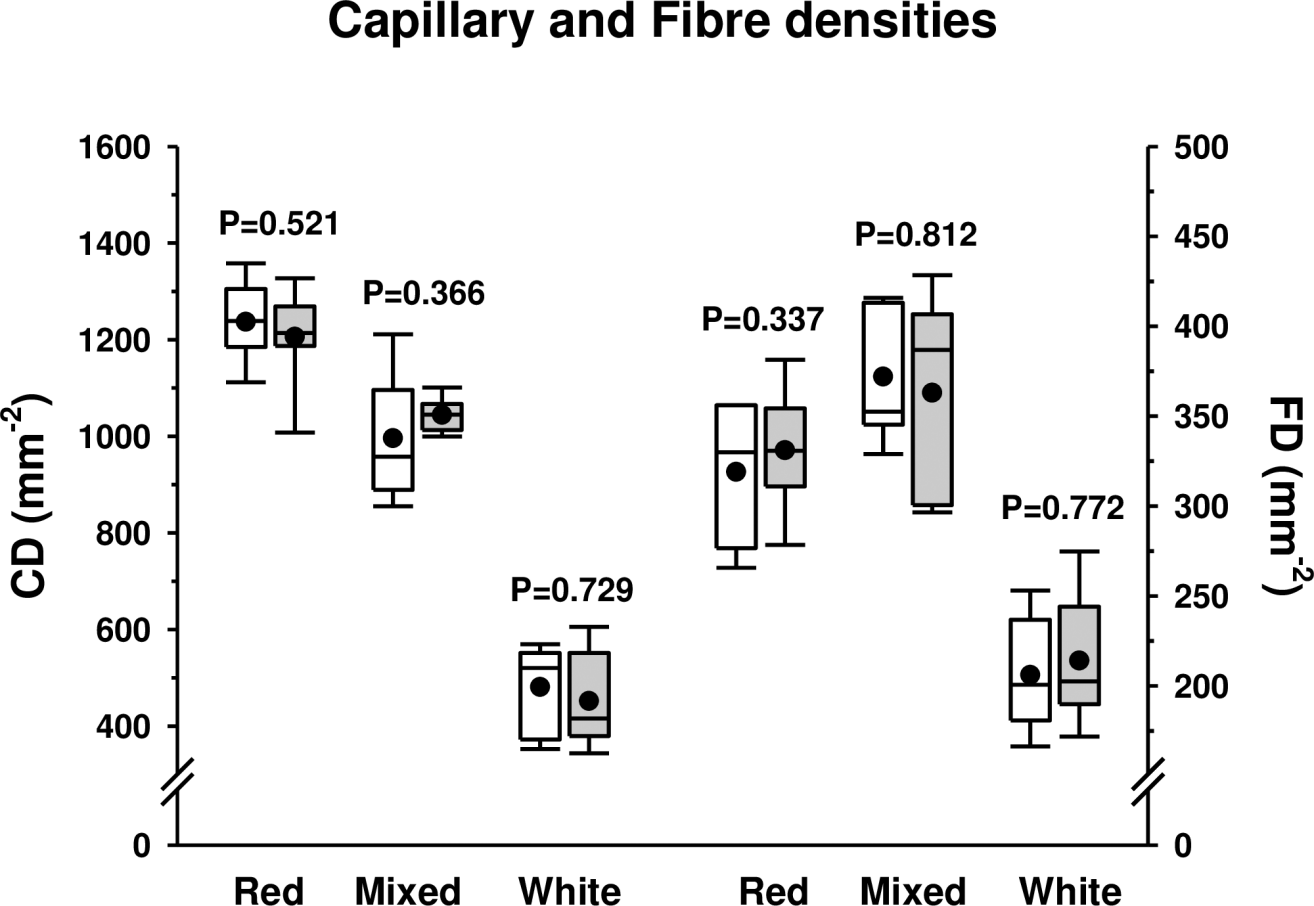
Capillary (CD) and fibre densities (FD) in red, mixed and white regions of the right and left rat gastrocnemius muscles. The boxes represent the first and third quartiles separated by the median, the mean is represented with a black dot, and the whisker end points represent the minimum and maximum values. Data from the right gastrocnemius are shown in white boxes and data from the left gastrocnemius in grey boxes. *p*‐values resulting from statistical comparisons between right and left muscles after running paired *t*‐Student tests are shown for each muscle zone on the graph

No contralateral significant differences were evidenced in either CD or FD in any region with *p*‐values ranging from 0.337 to 0.812 (Figure 5). A significant gradual decrease in capillary density (CD) from the red to the intermediate and white region was evident (*p* < 0.001 between all pairwise comparisons), indicating greater global regional capillarization in red and mixed than in white gastrocnemius. Figure 5 also shows significant lower fibre densities (FD) (*p* < 0.001 versus both red and mixed) in the white zone, reflecting the greater fibre sizes of the fibres from this region. The difference between red and mixed regions was also significant with *p* = 0.018.

### 3.4 Muscle force and fatigue parameters

**FIGURE 6:**
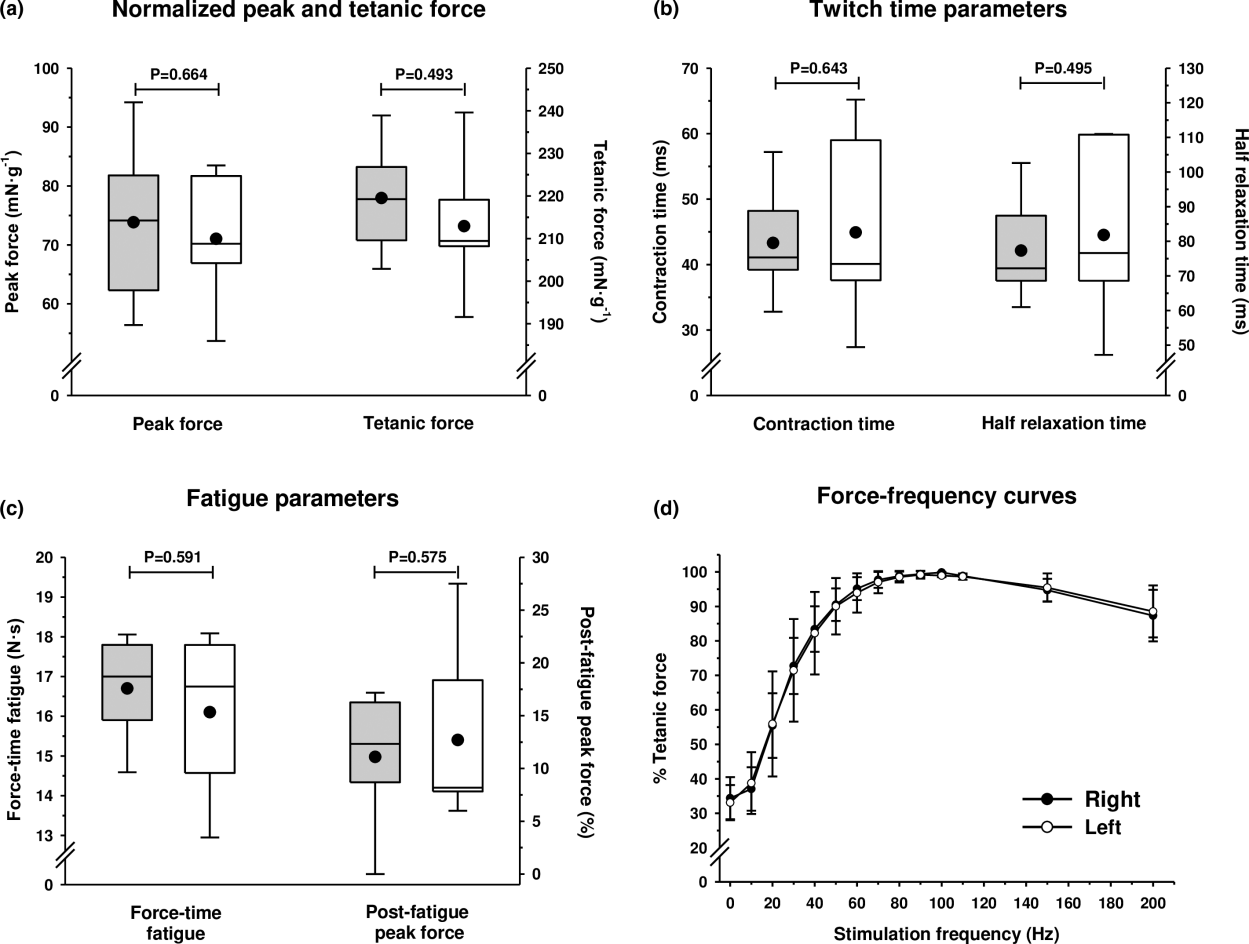
Force (a), twitch time (b) and fatigue parameters (c) in right and left rat gastrocnemius muscles. The boxes represent the first and third quartiles separated by the median, the mean is represented with a black dot, and the whisker end points represent the minimum and maximum values. Data from the right gastrocnemius are shown in white boxes and data from the left gastrocnemius in grey boxes. *p*‐values resulting from statistical comparisons between right and left muscles after running paired *t*‐Student tests are shown on the graph. (d) Force‐frequency curves for right and left rat gastrocnemius muscles expressed as a percentage of the tetanic force at different stimulation frequencies. Hollow and filled circles indicate the mean values and segments of standard deviation. All *p*‐values resulting from statistical comparisons between right and left muscles after running paired *t*‐student tests were *p* > 0.05

No statistical differences were shown between right and left gastrocnemius muscle mass (gastrocnemius/body mass) (right: 6.55 ± 0.40‰ vs. left: 6.49 ± 0.40‰, *p* = 0.265). Both right and left gastrocnemius muscles displayed similar mass‐normalized peak (*p* = 0.664) and tetanic force (*p* = 0.493) after sciatic stimulation (Figure 6a). This absence of significant differences was also evident for the twitch time parameters (contraction and half‐relaxation times) (Figure 6b) and the two fatigue parameters measured (force‐time and percentage of peak force after the fatigue test) (Figure 6c). Finally, the force‐frequency curves of right and left muscles, obtained after sciatic stimulation at gradually increased frequencies, almost totally overlapped indicating similar muscle force production at each stimulation frequency (Figure 6d). In all cases, the tetanic force was obtained at a mean frequency of 90–100 Hz.

## 4 DISCUSSION

The body and organs of animals can be divided relative to different planes, i.e., imaginary flat surfaces that pass through them. The sagittal plane is a vertical plane that divides the bodies or organs into right and left sides (Kardong, 2019). In humans, some studies have attempted to corroborate the existence of symmetry between muscles on the right and left sides of the lower limb. Thus, it has been found that vastus lateralis and soleus muscles did not present systematic differences between right and left legs neither in the fibre‐type proportion nor in the area of the fibre (Fugl‐Meyer et al., 1979; Horwarth et al., 2021; Lexell & Taylor, 1991; Tarnopolsky et al., 2007; Venturelli et al., 2021). Additionally, in the vastus lateralis, the absence of differences between both sides has also been reported in the number of myonuclei per fibre, the myonuclear domain, the transcriptome expression pattern and the isometric strength (Horwarth et al., 2021; Tarnopolsky et al., 2007). These studies have been carried out both in young adults (18–40 years) (Horwarth et al., 2021; Lexell & Taylor, 1991) and in older people (65 years) (Tarnopolsky et al., 2007), demonstrating that, despite age‐related changes in skeletal muscle, the symmetry between muscles of both sides of the body remains similar. However, some discrepancies between muscle symmetry have been observed in resistance‐trained men, who had differences between right and left vastus lateralis in fibre type proportion, although these differences did not translate into changes in strength (Arevalo et al., 2018; Lindström et al., 1995). This bilateral symmetry is not seen in athletes from unilateral sports either (Demura et al., 2010).

Regarding animal models, Lexell et al. (1994) analysed fibre type proportion and fibre density of right and left tibialis anterior and extensor digitorum longus in rabbits, finding no statistical differences between muscles of each leg. Later on, Rab et al. (2000) did not report differences between rabbit right and left rectus femoris, vastus medialis and adductor magnus muscles in fibre types and fibre areas. In rats, Guo and Zhou (2003) did not find differences between contralateral muscles in the concentration of some metabolites involved in lipid metabolism, such as glycerol, fatty acids and triglycerides, in muscles with mixed fibre composition (quadriceps, tibialis anterior and gastrocnemius), concluding that there is no metabolic laterality in the rat hind limb muscles (Guo & Zhou, 2003).

To the best of our knowledge, there are no histological or functional studies on rat gastrocnemius laterality. The gastrocnemius muscle is heterogeneous in its architecture, with three different regions within its cross‐section according to the fibre‐type proportion (Figure 1) (Armstrong & Phelps, 1984). Our results did not show statistical differences in fibre type composition between the right and left gastrocnemius in any of the three muscle regions (red, mixed and white) (Figure 3). All fibre types presented an almost identical fibre size (FCSA) in the right and left gastrocnemius, with this pattern maintained across the muscle section (Figure 4a–c), explaining the same fibre densities found on both sides (Figure 5). Similar individual fibre capillarity, as indicated by the CCA, and global muscle zone capillarization, estimated after the CD, are also evident (Figures 4d–f and Figure 5). Finally, SF (circularity) did not show significant differences between legs (Figure 4g–i). FCSA and SF depend on the accuracy of the cryostat cuts since these parameter's values could be altered with changes on the sample orientation in the cryostat and also due to the 3D muscle fibre architecture. The line of action between muscle insertion and its origin is different depending on the level where cuts were done due to differences in fibre length, pennation angle and curvature (Papenkort et al., 2020; Stark & Schilling, 2010; Wick et al., 2018). This intramuscular architecture entails different fibre morphometrical measurements along the proximal, equatorial or distal muscle level (Torrella et al., 2000). To avoid as much as possible biased results derived from changes in muscle orientation and muscle architecture characteristics, all muscles were cut in the same position at the equatorial (see Section 2), resulting in no changes observed in the FCSA and the SF of the fibres between right and left gastrocnemius muscles.

Taking into account that the different regions of the gastrocnemius muscle are recruited and used for different locomotion or postural activities (Kyröläinen et al., 2005), our results support the hypothesis that both gastrocnemius muscles are used equally for the same tasks or have a similar pattern of use.

It is well known that muscle force capacities are directly related to fibre type and muscle fibre size (Huard et al., 2002; Kardong, 2019). Thus, the absence of morphological differences between right and left gastrocnemius discussed above is in accordance with the lack of differences in muscle force production. All strength parameters measured in the present study were similar in the right and left gastrocnemius, showing no functional differences between legs (Figure 6). Moreover, as shown in Figure 6d, the same force‐frequency curves were observed in both muscles, suggesting that the Ca^2+^‐handling and cross‐bridge kinetics are similar. Since the structure and function are closely interdependent (Fugl‐Meyer et al., 1982), the morpho‐functional symmetry reported here lends additional support to a symmetrical use for both legs in the laboratory rat.

Thus, the morphological and physiological results derived from this study imply that, in further studies, one muscle could be used for intervention and the contralateral as a control. Moreover, right or left gastrocnemius could be used indistinctly for histological, functional and, presumably, biochemical determinations. This is relevant because, in studies aimed at obtaining a representative value of a given parameter for the gastrocnemius muscle, it will not be necessary to analyse both sides of the muscle, thus reducing the total number of assays needed for the experimentation. Furthermore, in research in which a large number of invasive experiments are carried out, which cannot all be applied in the same muscle, both contralateral muscles can be used, thus reducing the total number of animals (Böl et al., 2015). However, in some cases, further consideration is required and the use of the contralateral muscle as a control must be considered with caution in long‐term experiments. For example, in studies involving unilateral interventions that cause compensatory mechanisms in the neuromuscular system, as is the case of investigations inducing denervation of one side, hypertrophy and shifts in the fibres of the contralateral non‐intervened muscle have been described (Rab et al., 2000). Moreover, denervation, immobilization and reinnervation could alter the animal's locomotion pattern, producing changes in the afferent activity, which in turn is translated into alterations in efferent activity in the contralateral non‐intervened leg (Rab et al., 2000).

## 5 CONCLUSION

This is the first study in which morphological and functional lateral symmetry of the gastrocnemius muscle of the rat has been demonstrated. Our results report the absence of differences between right and left sides in muscle morphology (size and proportion of fibre types and muscle capillarization) and in muscle functional properties (twitch and tetanic force, twitch time parameters, fatigue parameters and force‐frequency curves). These findings make it possible to state that the use of the unilateral leg model for the study of interventions in the gastrocnemius muscle is the correct method since it can be assumed that both gastrocnemius had the same morpho‐functional profile before the intervention.

## AUTHOR CONTRIBUTIONS

Garoa Santocildes, Federica Fabiani, Marc Merino and Joan Ramon Torrella processed muscle samples and performed muscle force experiments; Federica Fabiani and Merino Marc acquired microphotographs, performed fibre typing and obtained fibre morphometrical data; Garoa Santocildes and Joan Ramon Torrella analysed and interpreted histochemical and muscle force data; Garoa Santocildes and Merino Marc wrote manuscript draft; Ginés Viscor, Teresa Pagès, Mario Marotta and Joan Ramon Torrella reviewed and edited the text; Garoa Santocildes, Ginés Viscor and Joan Ramon Torrella conceptualised and designed the study; Mario Marotta and Teresa Pagès supervised experiments and provided technical advice; Ginés Viscor, Mario Marotta, Joan Ramon Torrella, and Teresa Pagès dealt with the project administration.

## Data Availability

The data that support the findings of this study are available from the corresponding author upon reasonable request.
